# Quantitative Diagnostic Method for Biceps Long Head Tendinitis by Using Ultrasound

**DOI:** 10.1155/2013/948323

**Published:** 2013-12-07

**Authors:** Shih-Wei Huang, Wei-Te Wang

**Affiliations:** ^1^Department of Physical Medicine and Rehabilitation, Shuang Ho Hospital, Taipei Medical University, Taipei, Taiwan; ^2^Department of Physical Medicine and Rehabilitation, Changhua Christian Hospital, No. 135 Nan-Hsiao Street, Changhua, Changhua 500, Taiwan

## Abstract

*Objective*. To investigate the feasibility of grayscale quantitative diagnostic method for biceps tendinitis and determine the cut-off points of a quantitative biceps ultrasound (US) method to diagnose biceps tendinitis. *Design*. Prospective cross-sectional case controlled study. *Setting*. Outpatient rehabilitation service. *Methods*. A total of 336 shoulder pain patients with suspected biceps tendinitis were recruited in this prospective observational study. The grayscale pixel data of the range of interest (ROI) were obtained for both the transverse and longitudinal views of the biceps US. *Results*. A total of 136 patients were classified with biceps tendinitis, and 200 patients were classified as not having biceps tendinitis based on the diagnostic criteria. Based on the Youden index, the cut-off points were determined as 26.85 for the transverse view and 21.25 for the longitudinal view of the standard deviation (StdDev) of the ROI values, respectively. When the ROI evaluation of the US surpassed the cut-off point, the sensitivity was 68% and the specificity was 90% in the StdDev of the transverse view, and the sensitivity was 81% and the specificity was 73% in the StdDev of the longitudinal view to diagnose biceps tendinitis. *Conclusion*. For equivocal cases or inexperienced sonographers, our study provides a more objective method for diagnosing biceps tendinitis in shoulder pain patients.

## 1. Introduction 

Biceps tendinitis is the inflammation of the tendon around the long head of the biceps muscle. Acute biceps tendinitis may occur because of sudden overuse, especially among older patients (over 35 years of age for athletes and over 65 years for nonathletes) [1]. For shoulder pain patients, biceps tendinitis can be one of numerous etiologies and can accompany other pathologies of the shoulder [2]. Previous studies have listed the following biomechanical causes for biceps tendinitis: coracoacromial ligament thickening, impingement beneath the coracoacromial arch by a bone spur, and acromial apophysis unfusion [3, 4]. These pathologies can lead to biceps tendinitis because of repeated trauma by overuse and improper biomechanical circumstances. The inflammation process can initially lead to biceps tendon hyperemia and subsequent swelling of the tendon sheath because of interstitial tissue osmolarity that is changed by the release of chemokine. In the end stage of chronic inflammation, scarring and adhesion of the biceps tendon in the bicipital groove can occur. These symptoms can be obstacles to activities of daily living, and correct diagnosis and early treatment of biceps tendinitis are vital.

Numerous methods can be used for diagnosing biceps tendinitis. Patients with biceps tendinitis often complain of a deep, throbbing pain in the anterior shoulder that is intensified when lifting. The pain is usually localized to the bicipital groove and might radiate toward the insertion of the deltoid muscle. A history of occupational or sports overuse trauma could be the cause of biceps tendinitis in patients. In addition to obtaining the physical history of the patient, a physical examination in clinics can help us to differentiate biceps tendinitis from other possible causes of shoulder pain. Direct compression on the bicipital groove can induce tenderness, and this is the most common finding in biceps tenosynovitis patients. Other provocative tests are often used in evaluations, such as Speed's and Yergason's tests. The first evaluates the resistance force of the patient forearm by using a posture of shoulder flexion, elbow extension, and forearm supination; the second evaluates the forearm supination resistance by using elbow flexion. Both tests are defined as positive when the pain is provocative on the bicipital groove when using resistance force [5]. However, although clinical physical examinations are easily performed in clinics and can be helpful for biceps diagnosis, their sensitivity and specificity are insufficient for a precise diagnosis. According to Chen et al., Yergason's tests had a sensitivity of 32% and specificity of 78%, whereas Speed's tests had a sensitivity of 63% and specificity of 58% [6]. By contrast, ultrasound (US) examinations for rotator cuff injuries, which have been widely used since the 1980s, are more accurate. An US examination is also noninvasive and less expensive than magnetic resonance imaging (MRI) for evaluating soft tissue injuries but still offers the accuracy of a musculoskeletal US examination, which is comparable with MRI, and has a high accuracy in identifying rotator cuff injuries [7–11].

The US image is revealed by pixel echogenicity, which is reflected by different tissue contextures. The echogenicity cannot be precisely measured by visual evaluation alone. Quantitative evaluation of the grayscale histogram function of various ultrasonic machines has been applied to reveal the gray levels of pixel distributions in a certain range of interest (ROI). Furthermore, measures derived from the co-occurrence matrix (contrast, energy, and homogeneity) quantify the grayscale distribution in the expected direction of the fibrillar pattern in the tendon [12]. Healthy tendons exhibit alternating bands of light and dark because of reflections from well-organized collagen fibers in the US. Therefore, the grayscale pixels of the ROI represent the homogeneity or heterogeneity variations within the biceps tendon image in the US. In addition to postprocessing software, this ROI evaluation can also be performed in picture archiving and communication systems (PACS) [13–15].

US diagnosing sensitivity was 85.7%, and the specificity of detecting biceps tendon abnormalities was 98.3% (e.g., tendinosis, tenosynovitis, and tears) [16]. However, it is well-known that an US examination is highly operator dependent and requires expertise to improve the correctness of the diagnoses. Moreover, in clinical practice, disagreements over a biceps tendinitis diagnosis may result from equivocal cases. According to our research, no studies have outlined the quantitative diagnosis of biceps tendinitis by US using the echogenicity. Therefore, this study aims to fill this research gap by objectively investigating the cut-off values of the quantitative parameters of US to diagnose biceps tendinitis.

## 2. Methods

### 2.1. Participants

The institutional review board at our hospital approved the study and waived the necessity of obtaining patient consent. All data in our study were anonymized in this prospective observational study. Between August 2011 and April 2012, 379 patients with shoulder pain were recruited through the Physical Medicine and Rehabilitation outpatient department. All the participants were diagnosed with biceps tendinitis on the basis of their clinical presentation and physical examination (Speed's test or Yergason's test) in our clinic. The exclusion criteria were a history of shoulder joint surgery, fracture of the shoulder joint, recent local injection into the biceps tendon sheath, and calcification, tear, or rupture of the biceps tendon as detected by US. Demographic data, such as age, sex, body weight, and body height were obtained, and shoulder pain duration was also recorded in the clinic. In addition to shoulder pain participants, participants without shoulder pain symptoms were enrolled in the control group. In the ethic aspect, the database used consisted of deidentified secondary data. The data were analyzed anonymously and the need for informed consent was waived.

### 2.2. Ultrasound

The technique or protocol for evaluating the shoulder biceps tendon was adapted from that of Middleton [17]. The patient was seated in a chair in a neutral position with the elbow flexed to 90° and the forearm fully supinated in the palm up position. The greater and lesser tuberosities of the humerus bone were palpated to define the bicipital groove. The probe was placed on a level between the greater and lesser tuberosities for a transverse biceps tendon examination. The longitudinal view was obtained with the probe resting perpendicularly to the bicipital groove. To obtain a better image and eliminate the anisotropy effect, the probe was adjusted to be parallel to the tendon for both the transverse and longitudinal views.

All participants were examined within one week after being examined by experienced physiatrists who were qualified by the Taiwan Medical Ultrasound Association. The musculoskeletal US setting was as follows: an ACUSON Antares System (Siemens America, Inc.) linear-array probe (Siemens VFX13-5) with 5–12 MHz was focused to a depth of 1.5–2 cm. The depth-dependent gain setting was kept constant at 30 dB and the frequency was set to 10 MHz. The MSK mode using harmonic image processing of the tissue was selected for this study.

The diagnostic criteria for biceps tendinitis was defined as meeting at least one of the following: (1) tendon sheath swelling (transverse view: for women ≥4.6, for men ≥5.5 mm; longitudinal view: for women ≥2.5, for men ≥2.8 mm, as adopted from Schmidt et al.) and (2) tendon sheath fluid accumulation (abnormal hypoechoic or anechoic accumulation relative to the subdermal fat, although occasionally this could be isoechoic or hyperechoic) in intra-articular material that is displaceable and compressible and ≥3 mm, as adopted from Bruyn et al. [18]. In addition to the diagnostic criteria, increased color flow signals were recognized around the swollen biceps tendon as essential to a biceps tendinitis diagnosis. All the physiatrists involved with the musculoskeletal US examination reached a consensus on these diagnostic criteria for the purpose of avoiding operator-dependent misdiagnosis.

**Figure 1:**
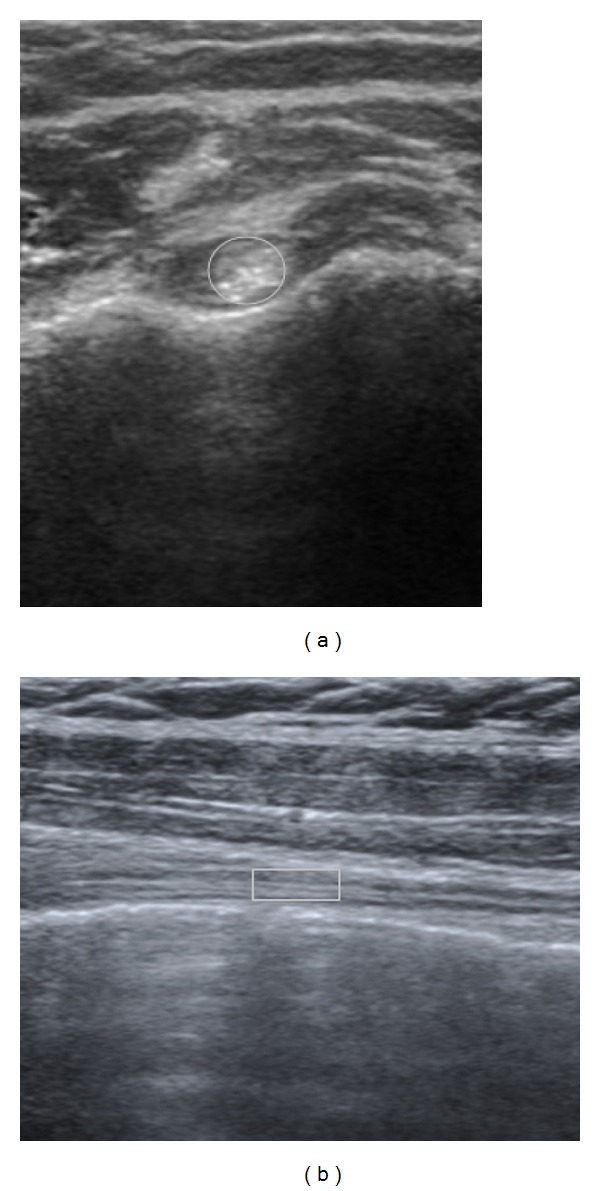
Biceps ultrasound. (a) Transverse view of the biceps tendon showing hypoechogenicity surrounding the bicipital tendon. The ROI was defined in a circular area, which covers the biceps tendon sheath. (b) The hypoechogenicity pattern among the biceps tendon is shown in this longitudinal view. The ROI was selected to be in the part of the biceps tendon with the most significant fluid accumulation, and the boarder part of this square area is defined as being located on the biceps tendon sheath. The grayscale values of the ROI were directly calculated by using the PACS system.

We adopted the quantitative echogenicity assessment method of Yu et al. for the biceps tendinitis evaluation [19]. The images were first uploaded to the PACS system in our hospital, from which the ROI data could be collected directly. A physician who was unaware of the results of the US examination evaluated the ROI data to exclude interobserver variation errors. Before this study, the physician who performed the ROI evaluation was trained in biceps anatomy and the basic musculoskeletal US evaluation method for one hour. The ROI was set at the center of the biceps tendon image within the focus area. A circular area of the ROI was applied to evaluate the transverse view of the bicipital tendon, and the margin was set at the sheath of the biceps tendon (Figure 1). A square area of the ROI was used for the longitudinal view of the biceps tendon. Both areas of the ROI were maintained at 2 mm^2^ and more than 2000 pixels were used. The echogenicity of the biceps tendon was revealed by the pixel intensity value (0 was black and 255 was white). The values of the minimum, maximum, and average of the ROI pixels were obtained for analysis. Furthermore, the standard deviation (StdDev) of the ROI pixels was recorded for heterogeneity representation.

### 2.3. Statistical Analysis

Continuous variables were represented as mean ± StdDev. We subdivided the shoulder pain participants into 2 groups according to whether they had positive biceps tendinitis or not, as determined by the consensus reached by the physiatrists using the diagnostic criteria. Independent *t*-tests and chi-square tests were applied to determine the significance of differences observed between the groups and control group between negative biceps tendinitis group. The ROI data were entered by bivariate logistic regression to predict biceps tendinitis.Discrimination was assessed using the area under the receiver operating characteristic (AUROC) curve. The AUROC curve was generated by plotting the sensitivity against one minus the specificity, and the area under the curve was calculated at a 95% confidence interval (CI). AUROC curve analysis was also employed to calculate the cut-off values, sensitivity, specificity, and overall correctness. Finally, the cut-off points were calculated by obtaining the best Youden index (sensitivity + specificity − 1). The SPSS version 20.0 for Mac (IBM Inc., USA) software was used for the statistical analyses, and a *P*  value < .05 was considered statistically significant.

## 3. Results

A total of 379 shoulder pain patients with an initial diagnosis of biceps tendinitis by physical examination were enrolled in this study. Of these, 43 were excluded: 17 were excluded because of biceps tendon tears or ruptures, 16 had a history of local injections before the US examination, and 10 had fractures or a history of operations. A total of 336 patients (aged 52.8 ± 13.4 years, 40.5% of which were men, and patients with body weights of 59.6 ± 11.9 kg, heights of 161.3 ± 8.0 cm, and symptom durations of 10.9 ± 9.5 wk) completed the US and ROI evaluations. Of these, 136 patients (aged 53.8 ± 11.9 old, 39.7% of which were men, and patients with body weights of 59.1 ± 11.2 kg, heights of 160.7 ± 7.4 cm, and symptom durations of 11.7 ± 9.4 wk) were diagnosed as having biceps tendinitis based on the diagnostic criteria for the US; the remaining 200 patients (aged 52.2 ± 14.3 years, 41.0% of which were men, and patients with body weights of 60.0 ± 12.3 kg, heights of 161.6 ± 8.4 cm, and symptom durations of 10.4 ± 9.6 wk) did not meet the diagnostic criteria for biceps tendinitis. There are 73 participants (aged 51.3 ± 13.8 years, 39.7% of which were men, and patients with body weights of 60.0 ± 13.3 kg, heights of 161.9 ± 7.7 cm) who were enrolled in the control group and the ultrasound protocol completed, too. When comparing the ROI values between the biceps tendinitis positive and negative groups, the independent *t*-tests indicated that there were significant differences in the maximal, minimal, and StdDev values of the grayscale pixels of the ROI for both the transverse and longitudinal views of the US (Table 1). When comparing bicep tendinitis negative group and control group, there is no significant different between these two groups.

**Figure 2:**
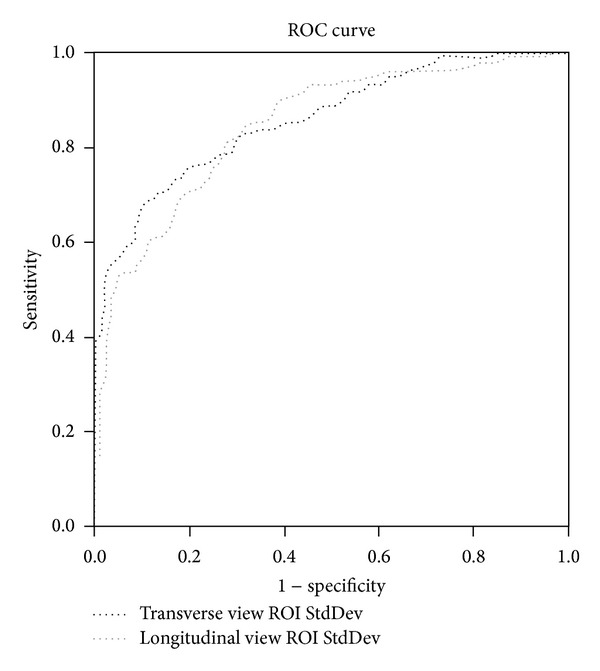
The area under the receiver operating characteristic (AUROC) curves depicting the sensitivity and specificity of biceps tendinitis by using the grayscale standard deviation (StdDev) values in the ROI from the transverse and longitudinal views.

Bivariate logistic regression analysis revealed that grayscale pixel values of the StdDev of the transverse ROI (odds ratio = 1.398, *P* < .001), the longitudinal ROI (odds ratio = 1.408, *P* < .001), and the minimal longitudinal ROI (odds ratio = 0.957, *P* = .028) are US predictors for biceps tendinitis (Table 2). AUROC curve analysis revealed that the minimal longitudinal ROI is unsuited for a cut-off point determination because the area was only 0.322. However, for the transverse and longitudinal views, the AUROC curves in the grayscale pixel StdDev values of the ROI were 0.856 and 0.852, respectively (Figure 2). We obtained the cut-off point of the grayscale pixel StdDev values of the ROI in the transverse view when it was greater than 26.85; the sensitivity was 68% and the specificity was 90% for biceps tendinitis. In addition to the transverse view, the longitudinal view cut-off point from the grayscale pixel StdDev values of the ROI can also be used, if it surpasses 23.25, and the sensitivity is 81% and specificity is 73% for biceps tendinitis (Table 3).

## 4. Discussion

Grayscale pixels of the ROI can represent the echogenicity of the US. They can reflect the character and condition of the examined tissue. In the biceps tendinitis group, more maximal StdDevs of the ROI for both the transverse and longitudinal views were observed than were observed in the nonbiceps tendinitis group. We assumed that inflammation causes interstitial fluid accumulation in the bicipital tendon sheath. The anatomical characteristics of the biceps tendon show that it is surrounded by the circular pattern of the tendon sheath. If the interstitial fluid accumulates in the tendon sheath, the swollen tendon sheath will compress the biceps tendon fibers. Therefore, the maximal echogenicity increases if the bicep fibers are condensed by compression from the swollen tendon sheath. It is reasonable that the minimal grayscale pixels of the ROI are fewer in the biceps tendinitis group from both transverse and longitudinal views because the echogenicity of water is hypoechoic. The sonographic heterogeneity of the ROI is represented by the StdDev value of the ROI. Our study reveals that the quantitative heterogeneity of sonography is more significant in the biceps tendinitis group. It is comparable to the pathology of inflammation of biceps tendons that results in altered echogenicity caused by tendinosis and interstitial fluid accumulation [9]. In addition, for the tendinosis morphology aspect, the attrition of the biceps tendon may appear thinned and frayed across the intertubercular sulcus of the humerus bone. The repeated friction of the biceps tendon against the irregular bone may progress to longitudinal splitting. The biceps tendon may be subdivided into 2 cords containing a central cavity. Heteroechogenicity can also be observed in the sonographic examination of tendinosis, and the different morphology pattern can help us differentiate from tendinitis. In the other words, the healthy biceps tendon presents a homogenous pattern under normal fibril alignment, and a lower StdDev value of the ROI can be expected [20]. Based on these findings, the grayscale pixels of the ROI in an US examination can be applied to an echogenicity evaluation of the biceps tendon.

US is generally perceived as an operator-dependent imaging modality. Even slight alterations in the probe or patient position may substantially change the appearance of a tendon. Operator dependence is frequently considered a limitation of US and is the most likely cause for this variation in the reported accuracy. Moreover, a previous study indicated that the poor agreement of US outcomes occurs when a marked disparity of the shoulder evaluation is associated with operator experience levels, and thus a training program is required for the precise detection of rotator cuff abnormalities [21]. Therefore, a quantitative method is useful to prevent a higher inaccuracy of diagnosing biceps tendinitis to counterbalance inadequate US experience, especially for equivocal cases. In our study, we found that the grayscale pixel StdDev values of the ROI for the transverse and longitudinal views in the US were predictors for biceps tendinitis. In equivocal cases of biceps tendinitis diagnosed using the criteria in Section 2, the transverse view of grayscale pixel StdDev values of the ROI offers a sensitivity of 68% and specificity of 90%, whereas for the longitudinal view of grayscale pixel StdDev values of the ROI, the sensitivity is 81% and specificity is 73%. Our results provide us with another method of diagnosis that has an objective and quantitative definition.

Our quantitative US diagnostic method is more accurate than physical examination in both sensitivity and specificity (Speed's test had a sensitivity of 63% and a specificity of 58%; Yergason's test had a sensitivity of 32% and a specificity of 78%) [6]. In addition, this method is comparatively easy to use. That is, as one study mentioned, for an experienced standard sonographer, high sensitivity (85.7%) and specificity (98.3%) can be achieved for biceps tendon lesion detection through MR arthrography, which is used as the gold standard examination; the sensitivity and specificity can also achieve 100% through a review by an experienced physician [16]. However, another study highlighting the poor interobserver and intraobserver reproducibility of biceps tendinitis stated that the overall agreement between MR arthrography and US when diagnosing bicep tendinitis was only 50% (positive agreement: 63%, negative agreement: 15%); Bruyn et al. concluded that this could be caused by a difference in US experience among the 14 sonographer participants [18]. Therefore, a quantitative US diagnostic method would be useful and is recommended in situations of inadequate US training and in disagreements over the diagnosis of biceps tendinitis.

Inflammation of the biceps tendon can influence the texture of the structure, and therefore can be represented by echogenicity. The histogram is considered to be a method that can be measured easily and objectively, and it was previously used for hepatic echo texture analysis [22]. The histogram is generally used to determine the distribution of the color intensity levels and can easily be evaluated using software available as part of the PACS program and based on the general use of the Digital Imaging and Communications in Medicine and PACS system. Therefore, in PACS, the color intensity within the ROI is shown on the monitor by an automatic calculation of the selected ROI. The histogram shows how the pixels in an image are distributed by graphing the number of pixels at each color intensity level. The grayscale pixel StdDev values represent the variation in the intensity values (darkest: 0, and brightest: 255), and they indicate the status of heterogeneity in the US signal. Our study proposes that the values can serve as objective parameters for diagnosing biceps tendinitis of patients with shoulder pain if they surpass the cut-off point.

Nevertheless, our study has the following limitations. First, various factors that influence echogenicity must be excluded. Prior research has indicated that less echogenicity is observed in rotator cuff tendons because of aging and minor tears [19]. To preempt this confounding factor, we excluded the patients with biceps tendon tears from our sample, and we demonstrated that no statistical differences existed between the demographic distributions of both groups. Furthermore, other studies have found no significant differences of echogenicity of the biceps tendon among different age groups [19]. Second, although the ROI was defined by only one physician to prevent interobserver errors, these images for the ROI were captured by different sonographers with different levels of experience. Although a previous study found that interobserver agreement for tendinitis of the biceps tendon is moderate, the operator-dependent factor cannot been completely dismissed [18]. Third, only one physician evaluated the ROI value. The interrater reliability of observers using this evaluation method was not examined in this study. To obtain a more objective quantitative study, we recommend further study of the interrater and intrarater reliability. Fourth, the gold standard diagnosis technique was not used in this study. Although the accuracy of US in the diagnosis of tendinopathy is generally accepted, arthroscopy and MRI still have an advantage over US in confirming biceps tendinitis. Finally, because this is a prospective observational study with differing criteria of inclusion and exclusion, certain confounding factors may still exist.

## 5. Conclusion

Sonographic grayscale pixels of the ROI for transverse and longitudinal views of the biceps tendon are available as quantitative tools for the diagnosis of biceps tendinitis. This method is more objective and relatively easy to use, especially for equivocal cases and situations involving inadequately experienced sonographers. Further studies are necessary to compare this method with shoulder MRI, which remains the gold standard of diagnosis. Moreover, the feasibility of using the US method to diagnose other rotator cuff lesions should be investigated in the future.

## Figures and Tables

**Table 1:** Demographic data and ultrasonic quantitative parameters among biceps tendinitis and nonbiceps tendinitis groups.

Parameters	Total (*n* = 336)	Biceps tendinitis (+) (*n* = 136)	Biceps tendinitis (−) (*n* = 200)	*P*
Age (year)	52.8 ± 13.4	53.8 ± 11.9	52.2 ± 14.3	0.272
Gender (male%)	40.5	39.7	41.0	0.822
BW (Kg)	59.6 ± 11.9	59.1 ± 11.2	60.0 ± 12.3	0.470
BH (cm)	161.3 ± 8.0	160.7 ± 7.4	161.6 ± 8.4	0.301
Duration (week)	10.9 ± 9.5	11.7 ± 9.4	10.4 ± 9.6	0.211
Transverse ROI				
Max.	191.5 ± 31.1	202.5 ± 28.0	184.0 ± 30.9	<0.001*
Min.	53.0 ± 21.0	45.1 ± 20.2	58.4 ± 19.9	<0.001*
Mean	112.0 ± 27.1	112.4 ± 24.9	111.8 ± 28.6	0.828
StdDev	25.2 ± 6.7	30.1 ± 6.8	21.9 ± 4.1	<0.001*
Longitudinal ROI	169.0 ± 35.9	180.0 ± 39.8	161.6 ± 30.9	
Max.	169.0 ± 35.9	180.0 ± 39.8	161.6 ± 30.9	<0.001*
Min.	44.9 ± 16.6	38.8 ± 16.0	49.1 ± 15.6	<0.001*
Average	97.7 ± 66.1	102.9 ± 99.2	94.2 ± 25.1	0.238
StdDev	21.9 ± 6.5	26.5 ± 6.0	18.7 ± 4.7	<0.001*

*P < 0.05 by independent student *t*-test.

**Table 2:** Quantitative parameters of biceps ultrasound for predicting biceps tendinitis.

Biceps ultrasound	*β*	SE	*R* ^2^	*P*	OR
Transverse ROI					
Max.	0.011	0.009	1.457	0.227	1.011
Min.	−0.009	0.025	0.124	0.725	0.991
Average	0.002	0.018	0.008	0.930	1.002
StdDev	0.335	0.068	24.518	<0.001*	1.398
Longitudinal ROI					
Max.	0.001	0.007	0.029	0.866	1.001
Min.	−0.044	0.020	4.844	0.028*	0.957
Average	−0.004	0.007	0.245	0.620	0.996
StdDev	0.342	0.057	36.257	<0.001*	1.408

*P < 0.05 by bivariant logistic regression.

**Table 3:** Accuracy of quantitative ultrasonic parameters for diagnosing biceps tendinitis.

Diagnostic parameters	AUROC ± SE	95% CI	*P*	Cut-off point	Youden index	Sensitivity (%)	Specificity (%)
Transverse ROI StdDev	0.856 ± 0.022	0.814–0.898	<0.001	26.85	0.58	68	90
Longitudinal ROI StdDev	0.852 ± 0.021	0.812–0.893	<0.001	21.25	0.53	81	73
